# Identifying and characterizing COPD patients in US managed care. A retrospective, cross-sectional analysis of administrative claims data

**DOI:** 10.1186/1472-6963-11-43

**Published:** 2011-02-23

**Authors:** Douglas W Mapel, Michael P Dutro, Jenő P Marton, Kimberly Woodruff, Barry Make

**Affiliations:** 1 Lovelace Clinic Foundation, Medical 2309 Renard Place SE, Suite 103, Albuquerque, NM, USA; 2 Medical Affairs, 235 E 42nd Street, Pfizer Inc, New York, NY, USA; 3 Global Health Economic and Outcomes Research, 235 E 42nd Street, Pfizer Inc, New York, NY, USA; 4 National Jewish Health, 1400 Jackson Street, Denver, CO, USA

## Abstract

**Background:**

Chronic obstructive pulmonary disease (COPD) is the fourth leading cause of death among US adults and is projected to be the third by 2020. In anticipation of the increasing burden imposed on healthcare systems and payers by patients with COPD, a means of identifying COPD patients who incur higher healthcare utilization and costs is needed.

**Methods:**

This retrospective, cross-sectional analysis of US managed care administrative claims data describes a practical way to identify COPD patients. We analyze 7.79 million members for potential inclusion in the COPD cohort, who were continuously eligible during a 1-year study period. A younger commercial population (7.7 million) is compared with an older Medicare population (0.115 million). We outline a novel approach to stratifying COPD patients using "complexity" of illness, based on occurrence of claims for given comorbid conditions. Additionally, a unique algorithm was developed to identify and stratify COPD exacerbations using claims data.

**Results:**

A total of 42,565 commercial (median age 56 years; 51.4% female) and 8507 Medicare patients (median 75 years; 53.1% female) were identified as having COPD. Important differences were observed in comorbidities between the younger commercial versus the older Medicare population. Stratifying by complexity, 45.0%, 33.6%, and 21.4% of commercial patients and 36.6%, 35.8%, and 27.6% of older patients were low, moderate, and high, respectively. A higher proportion of patients with high complexity disease experienced multiple (≥2) exacerbations (61.7% commercial; 49.0% Medicare) than patients with moderate- (56.9%; 41.6%), or low-complexity disease (33.4%; 20.5%). Utilization of healthcare services also increased with an increase in complexity.

**Conclusion:**

In patients with COPD identified from Medicare or commercial claims data, there is a relationship between complexity as determined by pulmonary and non-pulmonary comorbid conditions and the prevalence of exacerbations and utilization of healthcare services. Identification of COPD patients at highest risk of exacerbations using complexity stratification may facilitate improved disease management by targeting those most in need of treatment.

## Background

Chronic obstructive pulmonary disease (COPD) is the fourth leading cause of death among US adults and is projected to be the third by 2020 [1-3], although the disease is both preventable and treatable [4-6]. With this projected increased burden on healthcare systems, data describing how COPD patients are currently managed, together with information on COPD patients' healthcare utilization, are needed to inform healthcare organizations and providers. A small study of 1522 COPD patients in a health maintenance organization demonstrated that COPD patients had healthcare utilization and associated costs of more than twice those of age- and sex-matched controls [7]. Similarly, a larger study of over 100,000 patients aged ≥65 years with COPD or asthma showed the utilization of healthcare resources by these older COPD patients to be extremely high, both during hospitalization and after discharge [8]. The high prevalence of comorbidities in patients with COPD, especially respiratory conditions and cardiovascular disease, increases the morbidity associated with COPD [9-11].

Because of the burden imposed on healthcare systems and payers by patients with COPD, a means of identifying COPD patients who have higher healthcare utilization and associated higher costs is needed. The identification and stratification of COPD patients at risk of complications might facilitate management of these patients, improve care, and reduce costs. Furthermore, since COPD exacerbations, especially those requiring hospitalization, account for significant healthcare utilization, a method to accurately document COPD exacerbations using claims data would be very useful. Finally, comparing an older Medicare population with a working age commercial population will provide important information on how age influences the healthcare utilization in patients with COPD.

The purpose of this project is to describe a unique methodology that identifies COPD patients in a large managed care database and documents their demographics, comorbid conditions, and COPD exacerbations. We stratified these COPD patients by means of a novel algorithm of disease complexity (high, moderate, low complexity), used as a proxy of disease severity, and then examined the relationship between complexity of illness and key indicators of healthcare utilization and exacerbations. Furthermore, data were analyzed based on age group to determine if there are differences in COPD patients of an older Medicare (generally ≥65 years of age) population and a younger employer-based (< 65 years) population.

## Methods

### Study design and data source

This was a retrospective, cross-sectional analysis of US managed care administrative claims data from multiple health plans during the one-year study period, July 1, 2004, to June 30, 2005. The study population was extracted from a dataset of 12.4 million covered lives maintained by PharMetrics Inc (Watertown, MA, USA) from 19 health plans across the US: 3.2 million from the Northeast, 6.4 million from the Midwest, 1.8 million from the South, and 0.7 million from the West. The plans varied in size: 6 were <200,000 covered lives, 9 were between 200,001 and 1 million covered lives, and 4 were over 1 million covered lives. We evaluated the 7.79 million members who were continuously eligible during the study period for potential inclusion in the COPD cohort.

As differences likely exist between older and younger individuals, data from Medicare plans were evaluated separately from commercial health plans. The commercial population represented the employer-based managed care product offerings of Health Maintenance Organization, Preferred Provider Organization, and Point of Service plans. Due to the fragmentation of healthcare claims for the population aged older than 64 years, these patients were only included in Medicare analyses if they were continuously enrolled in a Medicare Risk product for the entire time span once becoming 65 years old.

### Identification of patients with COPD

Patients were identified as having COPD if they were aged ≥40 years and had *any *one of the following:

1. One inpatient hospitalization or one emergency room encounter with a COPD diagnosis (491.x [chronic bronchitis], 492.x [emphysema], or 496 [chronic airway obstruction]) listed in any position as a discharge diagnosis; or

2. Two professional claims, with different dates of services, with a COPD diagnosis listed in any position; or

3. A COPD-related surgical procedure (e.g. lung volume reduction) listed on either a professional or facility claim.

### Population demographics and comorbid conditions

The age and gender were determined for patients identified with COPD from claims data. Comorbid conditions were determined if claims data included diagnostic, procedures, and services codes (2004 ICD-9 CM, CPT-4, and HCPCS codes, respectively) for predetermined conditions (see Additional Files) during the reporting period. Condition frequencies were determined for respiratory and for non-respiratory comorbid conditions.

### Complexity

A claims-based classification of COPD complexity was created to serve as a surrogate for COPD disease severity. Comorbid respiratory conditions and medical procedures at any time during the study period were used to assign patients to one of three disease complexity levels (high, moderate, or low) based on selected diagnostic, procedures and services codes (2004 ICD-9, CPT-4, and HCPCS), as detailed in Additional File 1. Examples of code descriptions that resulted in a patient being classified as high complexity include a claim for cor pulmonale, tuberculosis, or malignant neoplasm. Examples for moderate complexity include pneumonia, cyanosis, bronchoscopy or dependence on supplemental oxygen. If a COPD patient did not have any comorbid condition for high or moderate complexity (Additional Files), they were classified as low complexity.

### Exacerbations

An algorithm was developed to identify and stratify COPD exacerbations using claims data (Table 1). Although there is controversy about the definition of a COPD exacerbation, the Global Initiative for Chronic Obstructive Lung Disease (GOLD) defines an exacerbation as: "*an event in the natural course of the disease *(COPD) *characterized by a change in the patient's baseline dyspnea, cough and/or sputum that is beyond normal day-to-day variation, is acute in onset, and may warrant a change in regular medication*" [1]. An exacerbation in this study was defined, as outlined in Table 1, by either a primary diagnosis recorded by a medical provider and coded in claims *or *a medication for an oral antibiotic commonly used for respiratory infection, or systemic steroid received by a patient during any 14-day time period. Exacerbation severity was classified by site of care reflecting resource utilization.

**Table 1:** Algorithm for identification and classification of exacerbations in COPD patients

*Criteria for exacerbations and exacerbation types (ranked in decreasing severity):*
**1. Inpatient hospitalized exacerbation**: an inpatient hospital stay with a primary diagnosis of COPD.
**2. Emergency room visit exacerbation**: an emergency room visit with a primary diagnosis of COPD.
**3. Ambulatory exacerbation identified by qualifying diagnosis**: an office or outpatient non-emergency room visit with any of the following diagnosis codes in the first position: 136.3, 466-466.19, 480-486, 487.0, 490, 491.21, 491.22, 493.02, 493.12, 493.22, 493.92, 494.1, 506.0-506.3, 507-507.8, 511.0-511.1, 512-512.8, 517.1, 518.0, 518.81, 518.82, 518.84, 770.84.
**4. Ambulatory exacerbation identified by qualifying drug therapy**: a pharmacy claim for the following oral antibiotics commonly used for respiratory infections amoxicillin, beta-lactamase inhibitors, second or third-generation cephalosporins, macrolides, or doxycycline) **or **a claim for systemic steroids (oral, intramuscular, or intravenous).

*Application:*

1. Only 1 exacerbation is attributed to a patient during any 14-day period (window).
2. The 14-day period starts with the first claim for an exacerbation of any type.
3. If the patient meets the criteria for >1 exacerbation during this 14-day window, only a single exacerbation of the most severe type is recorded.
4. A new 14-day period is begun when a new exacerbation of any type occurs outside of the previous 14-day window.

Total exacerbations included exacerbations of any severity (inpatient, emergency room, ambulatory by qualifying diagnosis, or ambulatory supported by drug therapy). Exacerbation frequency is reported for both total and hospitalized exacerbations as percent of patients experiencing ≥1 exacerbation(s), percent of patients experiencing multiple (≥2) exacerbations, and number of exacerbations/COPD patient.

### Utilization of healthcare services

The healthcare services assessed in this study included tests, procedures (including surgeries), and visits considered to be related with COPD (see Additional File 1). The most common or relevant of these are included in this report. These services were identified based on 2004 CPT-4 and HCPCS codes collected in claims. Hospital admissions and emergency room visits for any reason were identified based on the presence of a facility claim for an inpatient hospital stay or an outpatient emergency room visit.

### Data analysis

DTEC™software (Pfizer, New York, NY, USA, Version 3.3) was used to integrate administrative data and claims files, identify and stratify patients with COPD, as well as characterize demographics, comorbidities, and utilization of healthcare services. All of these analyses were specified prior to study initiation, and programmed in the software. These analyses, including the algorithms for COPD complexity stratification and exacerbation identification, were developed by a panel of experts that included pulmonologists, outcomes researchers and claims-based research consultants. They are based upon information from accepted guidelines [1,12] but also incorporate previous experiences of the panel in claims-based research. While DTEC™, a proprietary software program, was used for this analysis, the algorithms for COPD disease identification and stratification included in the software are specifically outlined and included in the body of this article or Additional File 1 so that they may be used in other claims querying systems.

Claims data during the 12-month study period were analyzed, and are presented as means with standard deviations. Categorical data are presented as numbers and percentages. Mean data were compared using the Student's *t*-test for normally distributed values and the Wilcoxon rank-sum test for non-normally distributed values. Categorical data were compared between commercial and Medicare populations before stratifying for complexity, using Chi-square test. Data were analyzed using GraphPad Prism^® ^statistical software (version 5.0 for Windows; GraphPad Software Inc., CA, USA). Because of large sample sizes a p-value of 0.01 was designated as being statistically significant for all comparisons. The database was compiled in accordance with all aspects of the Health Information Portability and Accountability Act (HIPAA) of 1996.

## Results

### Population characteristics

**Figure 1:**
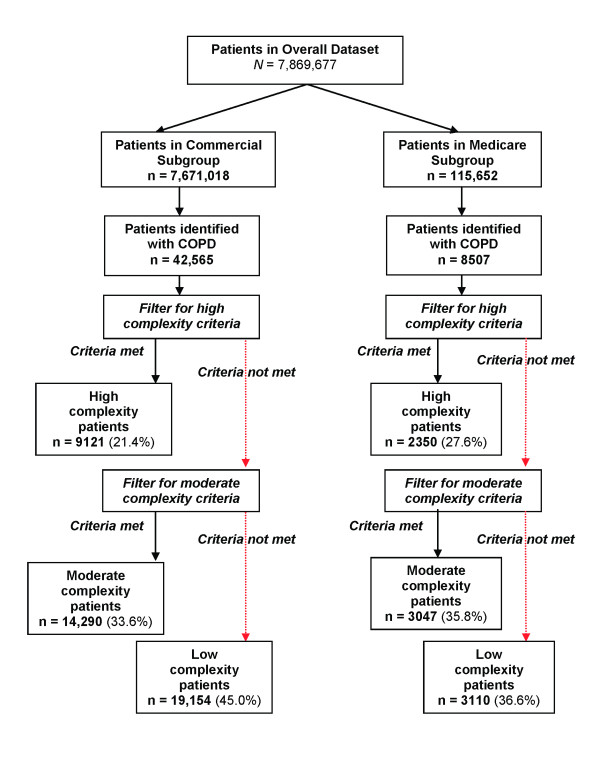
**Stratification of patients in the study**.

Eligible patients from the commercial or Medicare cohorts were identified as having COPD, and then stratified by complexity as shown in Figure 1. From the 7,671,018 health plan members in the commercial dataset, 42,565 (0.55%) met the criteria for COPD (Table 2). The median age was 56 years and 51.4% of patients were female. From the 115,652 health plan members in the Medicare dataset, 8,507 (7.4%) were identified as having COPD. In this dataset, the median age was 75 years and 53.1% were female. Although over half of both cohorts were classified with moderate- or high complexity disease, a larger proportion of the older Medicare cohort were classified as either high or moderate complexity (63.4%) compared with the younger commercial cohort (55.0%). This was mostly due to a higher percentage of high complexity patients in the Medicare cohort (Table 2).

**Table 2:** Demographic characteristics for the commercial and Medicare data sets, stratified by complexity

	Commercial dataset				Medicare dataset			
	
	All patients	Low complexity	Moderate complexity	High complexity	All patients	Low complexity	Moderate complexity	High complexity
	(n = 42,565)	(n = 19,154; 45.0%)	(n = 14,290; 33.6%)	(n = 9,121; 21.4%)	(n = 8,507)	(n = 3,110; 36.6%)	(n = 3,047; 35.8%)	(n = 2,350; 27.6%)
Parameter								
**Age, years**								
Median	56	56	57	57	75	75	75	75
**Age categories, n (% of column)**								
40-44 years	2,960 (7.0)	1,471 (7.68)	917 (6.42)	572 (6.27)	15 (0.2)	2 (0.1)	10 (0.3)	3 (0.1)
45-54 years	14,432 (33.9)	6,704 (35.0)	4,817 (33.7)	2,911 (31.9)	125 (1.5)	40 (1.3)	41 (1.3)	44 (1.9)
55-64 years	25,173 (59.1)	10,979 (57.3)	8,556 (59.9)	5,638 (61.8)	460 (5.4)	134 (4.3)	171 (5.6)	155 (6.6)
65-74 years	0 (0)	0 (0)	0 (0)	0 (0)	3,367 (39.6)	1,295 (41.6)	1,176 (38.6)	896 (38.1)
75-84 years	0 (0)	0 (0)	0 (0)	0 (0)	3,625 (42.6)	1,324 (42.6)	1,296 (42.5)	1,005 (42.8)
≥85 years	0 (0)	0 (0)	0 (0)	0 (0)	915 (10.8)	315 (10.1)	353 (11.6)	247 (10.5)
**Gender (%, of column)**								
Female	51.4	49.2	54.7	50.7	53.1	53.6	54.2	51.2

### Comparison of the commercial and Medicare COPD populations

Specific differences were identified in the prevalence of comorbid conditions between the commercial and Medicare data sets (Table 3). For example, most cardiovascular complications were significantly less common in the younger commercial cohort than the older Medicare cohort, including hypertension, ischemic heart disease, and heart failure. Those in the younger commercial cohort were significantly more likely to have episodes of upper respiratory tract complaints including allergic rhinitis and sinusitis compared with the older Medicare cohort, who were more likely to be diagnosed with pneumonia. Furthermore, a significantly higher proportion of patients in the younger commercial database compared with the older Medicare dataset were documented to have current tobacco use during the study period.

**Table 3:** Prevalence (%) of comorbid conditions in COPD patients from commercial and Medicare data sets*

	Commercial dataset (n = 42,565)	Medicare dataset (n = 8,507)
**Respiratory, %**		
Asthma	27.1	21.3
Sinusitis	21.2	8.4
Current tobacco use	19.0	7.5
Pneumonia	18.8	26.3
Allergic rhinitis	12.9	6.8
Sleep apnea	12.5	5.9
Respiratory failure	8.0	13.0
Pleurisy/pleural effusion	7.5	11.6
Pulmonary edema	5.7	11.2
Pulmonary heart disease	4.2	7.0
Respiratory tract cancer	4.2	7.1
**Non-respiratory, %**		
Hypertension	55.2	71.6
*Dyslipidemia*	*48.2*	*47.3*
Ischemic heart disease/angina	26.5	44.7
Diabetes	21.9	28.8
Heart rhythm conduction disturbance	20.4	38.9
Depression	14.5	9.4
Heart failure	14.4	33.8
Kidney disease/abnormal kidney function	11.5	21.9
Obesity	8.2	4.8
Cerebrovascular disease	9.2	20.5
Osteoporosis/decreased bone density	5.7	11.7
Myocardial Infarction (new or history)	4.1	7.1

*All of the differences comparing prevalence of comorbid conditions between commercial and Medicare populations were significant (p < 0.01), using two-tailed, chi-squared analysis, except for those indicated by italic text.

Most COPD patients in both the younger commercial cohort and the older Medicare cohort experienced at least 1 exacerbation during the study period (Table 4). Patients in the commercial cohort were more likely than Medicare members to experience an exacerbation of any type (commercial 70.6% and Medicare 61.0%), and the number of exacerbations per-patient in the commercial population was higher than in the Medicare population (2.13 vs. 1.58 per patient per year). However, patients in the older Medicare group were more likely than commercial patients to experience an exacerbation that led to hospitalization (19.4% vs. 13.9%). Furthermore, Medicare patients had more hospitalized exacerbations (0.25 per patient per year) than commercial patients (0.17 per patient per year). Multiple exacerbations (≥2) occurred more commonly in commercial patients (47.3% of commercial patients and 35.9% of Medicare patients), but multiple hospitalized exacerbations occurred in 2.3% and 3.6% of patients in each group, respectively.

**Table 4:** Prevalence (%) of patients experiencing ≥1 exacerbation and health services utilization among COPD patients in the commercial and Medicare datasets*

Parameter,	Commercial dataset (n = 42,565)	Medicare dataset (n = 8,507)
**Exacerbations**		

Inpatient hospitalization	13.9	19.4
*ER visit exacerbation*	*4.5*	*4.5*
Ambulatory exacerbations, with qualifying diagnosis	35.6	27.6
Ambulatory exacerbations supported by qualifying drug therapy	49.6	34.0
Total exacerbations (any of the above types)	70.6	61.0

**Health services**		

Hospitalization (any reason)	39.2	52.9
Office consultation/visit	98.7	96.4
Standard chest X-rays	73.1	74.8
Electrocardiogram	56.9	68.9
Pulmonary function testing	40.0	33.2
Respiratory-related equipment/supplies	26.0	35.7
Heart echo exam	25.8	40.1
Chest/thorax CT/MRI	23.6	26.6
*Cardiovascular stress test*	*19.2*	*19.2*
Respiratory/inhalation therapy	19.1	14.9
Cardiac catheterization/coronary angiography	9.3	8.0
*Bone density study*	*7.8*	*7.6*
Sleep studies	5.4	2.0
Disease management program	4.1	0
*Bronchoscopy*	*3.9*	*3.6*

*All of the differences comparing prevalence of comorbid conditions between commercial and Medicare populations were significant (p < 0.01), using two-tailed, chi-squared analysis, except for those indicated by italic text.

Important differences were identified in health service utilization between cohorts (Table 4). While nearly all of both populations experienced at least 1 office visit consultation, Medicare patients were significantly more likely to have been hospitalized for any reason than commercial patients. Although standard chest X-ray, ECG's, chest/thorax CT/MRIs, and heart echo exams were more likely in Medicare patients, more of the younger commercial patients had pulmonary function testing, cardiac catheterization/coronary angiography, and outpatient respiratory therapy. Only 39.0% of the total cohort had pulmonary function testing during the study period (40.0% commercial vs. 33.2% Medicare).

### COPD exacerbations and health service utilization by disease complexity

**Figure 2:**
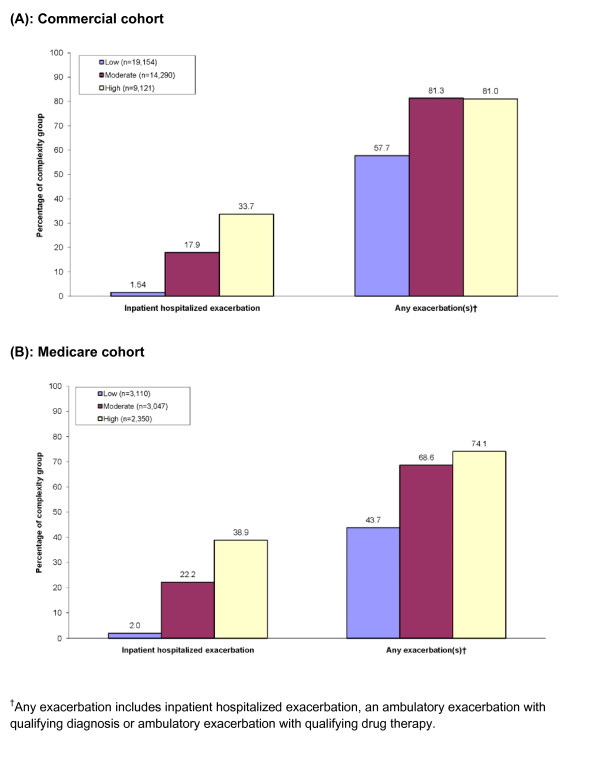
**COPD Patients Registering an inpatient exacerbation or experiencing any exacerbation, stratified by complexity, for (A) commercial, and (B) Medicare cohorts**. ^†^Any exacerbation includes inpatient hospitalized exacerbation, an ambulatory exacerbation with qualifying diagnosis or ambulatory exacerbation with qualifying drug therapy.

Evaluation of exacerbations by complexity group demonstrates a general increase in the percentage of patients experiencing total exacerbations and hospitalized exacerbations with increasing disease complexity in both age cohorts (Figure 2). In addition, a higher proportion of patients with high complexity disease in both data sets experienced multiple (≥2) exacerbations (61.7% high-complexity commercial patients; 49.0% high-complexity Medicare) than patients with moderate- (commercial 56.9%, Medicare 41.6%) or low-complexity disease (commercial 33.4%, Medicare 20.5%), highlighting the relationship between complexity and exacerbations. In addition, for both the commercial and Medicare data sets, high-complexity patients had higher numbers of exacerbations per patient (mean 3.08 commercial patients; 2.22 Medicare patients) compared with moderate (commercial 2.47; Medicare 1.76) or low (commercial 1.40; Medicare 0.91) complexity. The majority of patients experiencing multiple (≥2) exacerbations requiring hospitalization were classified as high complexity (7.9% high complexity commercial patients; 10.6% Medicare), with ≤2% of moderate-complexity patients (1.8% and 2.1%) and <1% of low-complexity patients (0% and 0.01%), respectively.

Utilization of healthcare services was also observed to increase with an increase in complexity (Table 5). High-complexity patients had more than twice the pulmonary function tests per-patient compared with low-complexity patients, in both data sets. Patients with high complexity illness had more office/consultation visits than those with low complexity illness (5.7 more office/consultations in the commercial group and 3.6 more in the Medicare data set).

**Table 5:** Proportion (%) of COPD patients in the Medicare and Commercial Cohorts Using ≥1 healthcare services, and mean number of each form of health service utilized per COPD patient, stratified by complexity of illness

	Commercial dataset			Medicare dataset		
	
Service, % (mean per patient)	Low complexity	Moderate complexity	High complexity	Low complexity	Moderate complexity	High complexity
	(n = 19,154; 45.0%)	(n = 14,290; 33.6%)	(n = 9,121; 21.4%)	(n = 3,110; 36.6%)	(n = 3,047; 35.8%)	(n = 2,350; 27.6%)
**Office consultation/visit**	**98.9**	**98.6**	**98.6**	**97.3**	**95.4**	**96.4**

Mean per patient	9.41	11.52	15.06	10.3	10.9	13.9

**Standard chest X-ray**	**62.7**	**77.5**	**88.0**	**63.1**	**74.5**	**90.9**

Mean per patient	1.25	2.45	6.37	1.3	2.4	6.3

**Electrocardiogram**	**50.6**	**56.5**	**71.0**	**60.7**	**66.3**	**83.1**

Mean per patient	1.14	1.57	2.63	1.6	2.2	3.9

**Pulmonary function**	**34.3**	**40.4**	**51.4**	**26.7**	**32.8**	**42.5**

Mean per patient	1.02	1.47	2.33	0.9	1.3	1.9

**Respiratory-related equipment/supplies**	**5.41**	**36.0**	**53.3**	**8.52**	**50.4**	**52.5**

Mean per patient	0.27	4.63	8.63	0.9	9.5	10.8

**Heart echo exam**	**18.4**	**25.5**	**41.9**	**29.4**	**37.6**	**57.7**

Mean per patient	0.68	1.06	2.00	1.1	1.5	2.8

**Chest/thorax CT/MRI**	**11.4**	**24.5**	**47.8**	**12.5**	**23.6**	**49.1**

Mean per patient	0.19	0.42	1.14	0.2	0.4	1.0

**Cardiovascular stress test**	**18.7**	**18.6**	**21.3**	**18.7**	**17.8**	**21.9**

Mean per patient	0.32	0.32	0.38	0.3	0.3	0.4

**Outpatient respiratory therapy services**	**10.3**	**25.7**	**27.1**	**4.95**	**20.6**	**20.7**

Mean per patient	0.16	0.69	1.00	0.1	1.3	1.4

**Cardiac catheterization/coronary angiography**	**7.48**	**8.92**	**13.8**	**5.05**	**7.68**	**12.4**

Mean per patient	0.51	0.61	0.94	0.3	0.5	0.8

**Sleep studies**	**1.72**	**4.43**	**14.7**	**0.35**	**1.51**	**4.68**

Mean per patient	0.03	0.08	0.31	0	0	0.1

**Bronchoscopy**	**0.32**	**2.88**	**13.2**	**0.16**	**2.0**	**10.1**

Mean per patient	0	0.07	0.35	0	0	0.2

## Discussion

This large, retrospective, cross-sectional analysis of US managed care administrative claims data describes a practical method to identify COPD patients using claims data. Furthermore, this methodology defines the complexity of their illness as a proxy of disease severity, and documents their exacerbations. The finding of a progressive increase in the prevalence of COPD exacerbations and utilization of health services for COPD patients from low to moderate to high complexity in both the commercial and Medicare data sets validates the utility of the complexity algorithm. Patients with COPD classified as high complexity had the highest health services utilization and were most likely to experience an exacerbation.

### Comorbid conditions

Our complexity classification was derived based on claims identifying selected comorbid conditions or medical procedures. Patients with COPD are typically thought to have comorbid diseases and conditions that contribute to the high burden of their disease [7,10,13,14]. Our data highlight that 4-27% of COPD patients had a respiratory comorbid condition, but up to 72% had a non-respiratory comorbid condition. Consequently over half of both age cohorts were stratified as moderate or high complexity. Our findings concur with other data [7,10], highlighting the high incidence of comorbidities in COPD patients. For example a study of 200 patients showed that patients with COPD had an average of 3.7 chronic medical conditions (including lung disease), compared with 1.8 chronic medical conditions for the controls [7]. Furthermore, a review by Sin et al., highlighted that a large proportion of patients with COPD have comorbid cardiovascular disease, depression, muscle wasting, reduced fat-free mass, osteopenia, and chronic infections [10].

Comorbidities in COPD patients contribute towards the high morbidity and mortality [14]. In particular, cardiovascular disease is a common cause of death in COPD patients [15-17], with co-prevalence estimates in COPD patients of 43% (mean age 68.8 years [14]) and 45% (mean age 67.5 years [7]). In the present study, similar prevalence estimates for key cardiovascular disease risk factors were found in commercial patients (median age 56 years), although higher prevalence in older patients, with 72% of the Medicare cohort (median age 75 years) having comorbid hypertension and 47% having dyslipidemia. In addition, heart failure (HF) is a risk factor for mortality in COPD patients, and particularly in those experiencing an exacerbation [14,18]. In the present study nearly 40% of the older Medicare cohort had HF, a proportion in agreement with a study of a similarly aged population among whom 40% had HF [18].

### Exacerbations

Our study describes a practical way to identify COPD exacerbations using claims data, and to classify them by site of care. In the current study the majority of patients, regardless of age, experienced COPD exacerbations, which often involved hospitalization or an emergency room visit. Others have used healthcare utilization to define the staging of COPD exacerbations, in order to incorporate those parameters considered most appropriate to base sub-classification [19]. Indeed, classification of an exacerbation as mild, moderate, or severe was deemed to be strongly related to the patient's underlying condition [19]. Accordingly, for a patient with severe COPD just a small change in lung function may present as a moderate-to-severe exacerbation because it necessitates physician intervention and increased healthcare utilization [19]. We stratified exacerbations by the site of care (inpatient hospitalization, emergency room visit, and ambulatory exacerbations), which enabled more detailed information regarding the patients' site of healthcare utilization to be accounted for. This is also somewhat similar to symptom-based classification of exacerbations often used in clinical trials which capture patients whose condition has changed enough to require a change in treatment, an emergency room visit, or hospitalization.

Patients in the older Medicare cohort were more likely to have moderate- or high complexity illness compared with the younger commercial dataset. This might be expected, as lung function and general health declines with age [20,21]. Our data also highlight that these patients qualified as high complexity were more likely to have multiple exacerbations and exacerbations requiring hospitalization compared with those of moderate or low complexity. As data were de-identified, it was not possible to confirm exacerbation information with corresponding medical records or clinical observations in this analysis. However, by distinguishing these high-risk patients from the overall COPD population, it might be ultimately possible to specifically target high-risk patients to limit the severity of their exacerbations with appropriate adjustment of therapy and/or monitoring of their comorbid conditions.

Although this study did not evaluate healthcare costs, the data generated could be used for future economic analyses and modeling. Others have reported the economic burden that exacerbations place on the healthcare system. Indeed, the estimated costs of exacerbations have been found to vary widely across studies from approx. $88 to $7,757 per exacerbation (2007 US dollars) [22]. Furthermore, exacerbations accounted for 35%-45% of the total per capita healthcare costs for COPD in one study, with costs increasing with severity of exacerbations [23]. Accordingly, investigating how actual or projected costs of COPD may change through identification of patients by exacerbations and subsequent stratification by complexity, as described in the present study, would be an interesting area to investigate further.

### Utilization of healthcare services

We identified groups of COPD patients that are high users of healthcare services. For example, over half of the Medicare COPD patients and almost 40% of commercial COPD patients were hospitalized at least once for any reason in a 1-year period. Evidence of high outpatient utilization is characterized by the finding that virtually all patients in both populations (≥96%) had at least 1 office visit/consultation with a mean of over 10 per patient during the year. Furthermore, our method of stratifying patients found differential healthcare utilization; our high complexity patients had the highest health services utilization across almost all of the services monitored. As our method of stratifying patients to high and medium complexity was dependent on their comorbid conditions and the procedures they received, it is logical to expect that these patients had higher utilization compared with those of low complexity.

We do not know if our high complexity group had higher healthcare utilization in subsequent years, and this would be an important subject for future investigations. Interestingly, although GOLD guidelines state that lung function testing with spirometry is essential for the diagnosis and management of COPD, and indeed can provide a useful description of the severity of pathological changes in COPD [1], less than half of the population had a pulmonary function test carried out during the study year.

Our data should be interpreted in light of some important limitations. These analyses are retrospective and descriptive in nature; there were no control groups. The large sample sizes resulted in statistically significant differences between cohorts that may not be clinically significant. However, we adjusted for the large sample size by defining significance as p < 0.01, rather than the conventional level of p < 0.05. Administrative claims data are primarily generated for reimbursement purposes rather than research purposes. Consequently, the accuracy of claims data is dependent on the precision and timing of the coding associated with their use. As such, some comorbid conditions, such as obesity and tobacco use, are often under-reported in claims data and likely are under-reported here as well. Furthermore, confusion over the differential diagnosis of asthma and COPD could have led to some patients in our COPD cohorts who actually had asthma rather than COPD. In fact, at least 1 claim with a diagnosis of asthma was recorded in 27% and 21% of the commercial and Medicare COPD cohorts, respectively. Findings of cross-sectional studies have shown a similar overlap of up to 30% between people who have a clinical diagnosis of COPD and asthma [24]. While some of these claims could represent coexisting asthma and COPD, the potential for diagnosis coding errors needs to be taken into consideration. The possibility that other respiratory conditions, such as bronchiectases, hypoventilation-obesity, or overlap syndrome, were miscoded as COPD should also be considered. Furthermore, there is a possibility that asthma attacks were miscoded as COPD exacerbations. Because the study period was limited to 1 year, it is possible for the data to provide false positives or negatives in the population selection criteria and overstate or understate the clinical severity of the disease being considered. In this case, individuals with COPD who did not have COPD-related claims within the observation period are not included in the analysis. Since these patients are likely to have less severe disease, this would result in the overall prevalence of COPD being understated, while the burden per patient with COPD overstated. On the other hand, because patients had to be continuously enrolled during the entire study year to be eligible for inclusion in this cross-sectional analysis, those who either left their health plan or died were excluded from the analysis. Excluding those who died could have removed those who were most ill and utilized the most healthcare resources. This could be especially important in the elderly cohort.

It is also possible that differences in exacerbations and healthcare utilization between different groups could be due to unrecognized confounders, such as duration of COPD, current smoking status, and type and duration of medication use. In addition, given that the study was a cross-sectional analysis, no cause and effect analyses could be conducted. We broadly compared an older Medicare population with a younger employer-based commercial population, although 7% of the Medicare population was <65 years and we were unable to extract these patients from the data set. However, as over 90% of the patients were ≥65 years we feel our observations provide useful information on how age influences the burden of COPD on managed care resources

Despite these limitations, the methodology presented here provides a practical way for healthcare providers to identify and stratify COPD patients and identify those experiencing exacerbations within a large managed care database. In turn, this might help healthcare providers prioritize patients at risk for future exacerbations and resource utilization, to help ensure that those COPD patients with the greatest need for close monitoring receive optimal care.

## Conclusions

We present a unique and practical method for identifying patients with COPD, determining disease severity - as "complexity of illness" - and documenting exacerbations using claims data. Our data highlight important differences in comorbidities, exacerbations, and healthcare utilizations in older (aged ≥65 years) compared with younger (aged <65 years) COPD patients. Furthermore, by stratifying COPD patients based on diagnostic, procedures, and services codes, we have demonstrated that patients stratified as having high- or moderate complexity disease experienced a higher number of exacerbations than those with low complexity disease. Additionally, by stratifying by complexity of illness we show linearity between complexity of illness and utilization of healthcare services and hospitalizations.

Identification of COPD patients at higher risk of complications using complexity stratification and/or identifying those experiencing exacerbations may serve to improve patient management, and ultimately reduce the burden of disease to the patient, and to the healthcare systems supporting them.

## Competing interests

Dr. Mapel was a paid consultant to Pfizer Inc in connection with conduct of the analysis and development of the manuscript. Dr. Mapel has served as a consultant to and received research funding from Pfizer Pharmaceuticals, GlaxoSmithKline, and AstraZeneca. Drs. Woodruff, Marton, and Dutro, are employees of, and own stock in Pfizer Inc. Dr. Make has participated in advisory boards and received honoraria for speaking from Pfizer Inc within the past five years.

## Authors' contributions

All authors participated in the design of the study, and contributed to drafting the manuscript. All authors read and approved the final manuscript.

## Disclosure

Parts of these data were presented as a poster at the American Thoracic Society 103^rd ^International Conference, May 22, 2007, San Francisco, CA, USA.

## Pre-publication history

The pre-publication history for this paper can be accessed here:

http://www.biomedcentral.com/1472-6963/11/43/prepub

## Supplementary Material

Additional file 1 **Classifying COPD patients by complexity of illness**. 1A: The analysis included the following comorbid conditions and health care services that were predetermined and evaluated by the presence of diagnostic, procedures, and services codes (2004 ICD-9 CM, CPT-4, and HCPCS codes). Diagnosis codes used to define COPD complexity level. 1B: Comorbid respiratory conditions and medical procedures at any time during the 1-year study period (July 1, 2004, to June 30, 2005) (see Additional File 1A) were used to assign patients to 1 of 3 disease complexity levels (high, moderate, or low) based on selected diagnostic, procedures and services codes (2004 ICD-9, CPT-4, and HCPCS), as detailed. If a COPD patient did not have any comorbid condition for high or moderate complexity, they were classified as low complexity. Click here for file
