# Supplementary material for: Identifying and characterizing COPD patients in US managed care. A retrospective, cross-sectional analysis of administrative claims data
Source: BMC Health Serv Res. 2011 Feb 23;11:43. doi: 10.1186/1472-6963-11-43 (PMC3050697; doi:10.1186/1472-6963-11-43)
Supplement: Additional file 1 — Classifying COPD patients by complexity of illness. 1A: The analysis included the following comorbid conditions and health care services that were predetermined and evaluated by the presence of diagnostic, procedures, and services codes (2004 ICD-9 CM, CPT-4, and HCPCS codes). Diagnosis codes used to define COPD complexity level. 1B: Comorbid respiratory conditions and medical procedures at any time during the 1-year study period (July 1, 2004, to June 30, 2005) (see Additional File 1A) were used to assign patients to 1 of 3 disease complexity levels (high, moderate, or low) based on selected diagnostic, procedures and services codes (2004 ICD-9, CPT-4, and HCPCS), as detailed. If a COPD patient did not have any comorbid condition for high or moderate complexity, they were classified as low complexity. [file 1472-6963-11-43-S1.PDF]

Additional Files

Additional File 1A: Definitions used to identify comorbid conditions and services used

The analysis included the following comorbid conditions and health care services that were predetermined and evaluated by the presence of diagnostic, procedures, and services codes (2004 ICD-9 CM, CPT-4, and HCPCS codes).

Comorbid conditions evaluated including definitions	
**Respiratory tract comorbid conditions**	asthma (excluding status asthmaticus) (base on dx only); respiratory failure; pulmonary edema; status asthmaticus; anoxia (asphyxia); current tobacco use; allergic rhinitis/hay fever; sinusitis; otitis media; upper respiratory infection; acute pharyngitis/tonsillitis (incl. streptococcal); tonsils/adenoids disease; acute bronchitis (excl. unspecified bronchitis); unspecified bronchitis; pneumonia; bronchiectasis; pneumothorax (spontaneous); pleurisy/pleural effusion; lung cancer; respiratory tract cancer (except lung cancer); cystic fibrosis; acute pulmonary heart disease; chronic pulmonary heart disease; aspergillosis; sleep apnea; other respiratory disease; chest pain (excluding angina pectoris); shortness of breath; dysphonia; throat irritation
**Non-respiratory tract comorbid conditions**	adverse effect of medicine; adrenal insufficiency/crisis/suppression; allergic reaction; MI or history of MI; cachexia/malnutrition; candidiasis of the mouth; cataract; cerebrovascular disease (incl. stroke); dementia/Alzheimer's/other cognitive disorder; depression (non-psychotic and/or psychotic/major); diabetes; dyslipidemia; ecchymosis; fracture of hip or vertebra(e); glaucoma; headache; heart failure; heart rhythm/conduction disturbance; hepatitis (drug-induced, toxic, non-infectious); HIV infection including aids; hypertension; hypotension; insomnia (excluding sleep apnea); ischemic heart disease/angina; liver transplantation status; kidney disease/abnormal kidney function; myopathy/muscle weakness; nausea/vomiting; non-ulcer peptic disease (e.g., heartburn, GERD); obesity; osteoporosis/decreased bone density; peripheral vascular disease; polycythemia; seizure; skin texture change (incl. thinning); sleep disturbance (excluding insomnia & sleep apnea); steroid toxicity; substance use disorder (including alcohol); theophylline toxicity; tremor; urinary retention; xerostomia (dry mouth)

Healthcare services evaluated (CPT and HCPCS descriptions)
transportation/ambulance; respiratory (incl. oxygen)-related equipment & supplies; steroid drug in/from office (not by prescription); antibiotic drug in/from office (not by prescription); sinus surgery, open; sinus and nasal surgery, endoscopic; larynx surgery; emergency intubation; laryngoscopy, diagnostic & therapeutic; trachea & bronchi procedures; bronchoscopy; thoracentesis/tube thoracotomy; lung volume reduction surgery; lung bullectomy (excision-plication); lung transplantation; chest/lung surgery, other; lung/pleural biopsy, closed; thoracoscopy, other; upper gastrointestinal endoscopy; sinus X-rays; chest X-rays, standard; chest X-rays, other than standard; CT/MRI: chest/thorax; upper gastrointestinal X-rays; vascular diagnostic radiology; chest ultrasound exam; bone density study; heart imaging, perfusion & nuclear; lung nuclear scan; chem./metabolic panel testing; theophylline assay; drug assay, quantitative, other; thyroid testing, excluding TRH/TRF; urinalysis; alpha-1-antitrypsin assay; carbon monoxide assay; sweat testing; gamma-globulin assays; blood gases determination; eosinophil blood cell count; CBC and component counts; infectious agent identification; skin testing, excluding tuberculosis; tuberculosis skin testing; streptococcal testing; stain/smear of bacteriological specimen; blood culture; bacterial culture, other; nose/throat culture; fungus exam/culture; antimicrobial susceptibility; virus identification; cytopathology (non-genital); eosinophil nasal smear; office e/m visit/consultation (count of CPT codes); home E/M visit or other home service; hospital e/m visit/consultation (count of CPT codes); critical care service; nursing facility E/M visit/service (CPT code count); immunization: influenza virus; immunization: pneumococcal; intravenous infusion; eye examination; heart/lung resuscitation (CPR); electrocardiogram (ECG); cardiovascular stress test; heart echo exam; cardiac catheterization/coronary angiography; pulmonary function testing; bronchoprovocation (challenge) testing; respiratory/inhalation therapy (incl. mechanical ventilation); pulmonary rehabilitation program; allergy (skin) testing; allergy immunotherapy; sleep studies; disease management program; smoking cessation treatment (count of CPT/HCPCS codes); health education; prolonged service/standby; emergency e/m visit (count of CPT codes); care oversight/case management; preventive medicine counseling

Additional File 1B: Diagnosis codes used to define COPD complexity level

Comorbid respiratory conditions and medical procedures at any time during the 1-year study period (July 1, 2004, to June 30, 2005) (see **Additional File 1A**) were used to assign patients to 1 of 3 disease complexity levels (high, moderate, or low) based on selected diagnostic, procedures and services codes (2004 ICD-9, CPT-4, and HCPCS), as detailed. If a COPD patient did not have any comorbid condition for high or moderate complexity, they were classified as low complexity.

**Diagnoses (ICD-9-CM code) used to define COPD complexity levels***	
**High complexity**	pulmonary TB (011); TB of lung, infiltrate (011.0); TB lung infiltrate -unspecified (011.00); TB lung infiltrate-no exam (011.01); TB lung infiltrate -exam unknown (011.02); TB lung infiltrate -microscopy dx (011.03); TB lung infiltrate -culture dx (011.04); TB lung infiltrate-histology dx (011.05); TB lung infiltrate-other test (011.06); TB of lung, nodular (011.1); TB lung nodular-unspecified (011.10); TB lung nodular-no exam (011.11); TB lung nodule-exam unknown (011.12); TB lung nodular-microscopy dx (011.13); TB lung nodular-culture dx (011.14); TB lung nodular-histology dx (011.15); TB lung nodular-other test (011.16); TB of lung with cavitation (011.2); TB lung with cavity-unspecified (011.20); TB lung with cavity-no exam (011.21); TB lung cavitation-exam unknown (011.22) TB lung with cavitation-microscopy dx (011.23) TB lung with cavitation-culture dx (011.24); TB lung with cavitation -histology dx (01125); TB lung with cavitation-other test (011.26); tuberculosis of bronchus (011.3); TB of bronchus-unspecified (011.30); TB of bronchus-no exam (011.31); TB of bronchus-exam unknown (011.32); TB of bronchus-microscopy dx (011.33); TB of bronchus-culture dx (01134); TB of bronchus-histology dx (011.35); TB of bronchus-other test (011.36); TB bronchiectasis (011.5); TB bronchiectasis-unspecified (011.50); TB bronchiectasis-no exam (011.51); TB bronchiectasis-exam unknown (011.52); TB bronchiectasis-microscopy dx (011.53); TB bronchiectasis-culture dx (011.54); TB bronchiectasis-histology dx (011.55); TB bronchiectasis-other test (011.56); tuberculous pneumonia (011.6); TB pneumonia-unspecified (011.60); TB pneumonia-no exam (011.61); TB pneumonia-exam unknown (011.62); TB pneumonia-microscopy dx (011.63) TB pneumonia-culture dx (011.64) TB pneumonia-histology dx (011.65); TB pneumonia-

	other test (011.66); tuberculous pneumothorax (011.7); TB pneumothorax-unspecified (011.70); TB pneumothorax-no exam (011.71); TB pneumothorax-exam unknown (011.72); TB pneumothorax-microscopy dx (011.73); TB pneumothorax-bacterial culture dx (011.74); TB pneumothorax-histology dx (011.75); TB pneumothorax-other test (011.76); pulmonary TB nec (011.8); other pulmonary TB unspecified (011.80); pulmonary TB no bacteriological or histological examination (011.81); other pulmonary TB bacteriological or histological examination unknown (011.82); other specified pulmonary TB tubercle bacilli found (in sputum) by microscopy dx (011.83); other specified pulmonary TB tubercle bacilli not found (in sputum) by microscopy but found by bacterial culture dx (011.84); other specified pulmonary tuberculosis tubercle bacilli not found by bacteriological examination but tuberculosis confirmed histology dx (011.85); Other specified pulmonary TB tubercle bacilli not found by bacteriological or histological examination but tuberculosis confirmed by other methods (011.86); tuberculous pleurisy (012.0); aspergillosis (117.3); pneumocystosis (136.3); malignant neoplasm trachea/lung (162); malignant neoplasm trachea (162.0); malignant neoplasm main bronchus (162.2); malignant neoplasm upper lobe lung (162.3); malignant neoplasm middle lobe lung (162.4); malignant neoplasm lower lobe lung (162.5); malignant neoplasm of other parts of bronchus or lung (162.8); malignant neoplasm of bronchus and lung unspecified (162.9); malignant neoplasm pleura (163); malignant neoplasm parietal pleura (163.0); malignant neoplasm visceral pleura (163.1); malignant neoplasm of other specified sites of pleura (163.8); malignant neoplasm pleura not specified (163.9); second malignant neoplasm pleura (197.2); cystic fibrosis (277.0); cystic fibros without meconium ileus (277.00); cystic fibrosis with pulmonary (277.02); cystic fibrosis with gastrointestinal (277.03); cystic fibrosis with other manifestations (277.09); secondary polycythemia (289.0); acute pulmonary heart disease (415); acute cor pulmonale (415.0); pulmonary embolism/infarct (415.1); iatrogenic pulmonary embolism and infarction (415.11); other pulmonary embolism and infarction (415.19); chronic pulmonary heart disease (416); primary pulmonary hypertension (416.0)
**Moderate complexity**	kyphoscoliotic heart disease (416.1); other chronic pulmonary heart disease (416.8); chronic pulmonary heart disease unspecified (416.9); SARS-associated viral pneumonia (480.3); pneumococcal pneumonia (481); other bacterial pneumonia (482); klebsiella pneumoniae (482.0); pseudomonal pneumonia (482.1); hemophilus influenzae pneumonia (482.2); streptococcal pneumonia (482.3); streptococcal pneumonia not specified(482.30); streptococcal group A pneumonia (482.31); streptococcal group B pneumonia (482.32); streptococcal pneumonia other (482.39); staphylococcal pneumonia (482.4); pneumonia due to staphylococcal unspecified (482.40); pneumonia due to staphylococcus aureus (482.41); other staphylococcus pneumonia (482.49); bacterial pneumonia other specified (482.8); pneumonia due to anaerobes (482.81); *escherichia coli* pneumonia (482.82); gram

negative pneumonia other unspecified (482.83); Legionnaires' disease (482.84); bacterial pneumonia other unspecified (482.89); bacterial pneumonia not specified (482.9); pneumonia: organism other specified (483); Chlamydia pneumonia (483.1); pneumonia: organism other specified (483.8); pneumonia in other infectious disease (484); pneumonia with cytomegalic inclusion disease (484.1); pneumonia in whooping cough (484.3); pneumonia in anthrax (484.5); pneumonia in aspergillosis (484.6); pneumonia in other system mycoses (484.7); pneumonia in infectious disease classified elsewhere (484.8); influenza with pneumonia (487.0); obstructive chronic bronchitis with acute exacerbation (491.21); obstructive chronic bronchitis with acute bronchitis (491.22); emphysematous bleb (492.0); extrinsic asthma with status asthma (493.01); extrinsic asthma with acute exacerbation (493.02); Intrinsic asthma with status asthmaticus (493.11); intrinsic asthma with acute exacerbation (493.12); chronic obstructive asthma with status asthmaticus (493.21); chronic obstructive asthma with acute exacerbation (493.22); asthma with status asthmaticus (493.91); unspecified asthma with acute exacerbation (493.92); bronchiectasis (494); bronchiectasis without acute exacerbation (494.0); bronchiectasis with acute exacerbation (494.1); extrinsic allergic alveolitis (495); Farmers' lung (495.0); allergic alveolitis and pneumonitis not specified (495.9); asbestosis (501); Pneumonitis due to solids and liquids (507); food/vomit pneumonitis (507.0); empyema (510); empyema with fistula (510.0); empyema without fistula (510.9); Pleurisy with effusion with a bacterial cause other than TB (511.1); pneumothorax (512); spontaneous tension pneumothorax (512.0); spontaneous pneumothorax other (512.8); abscess of lung and mediastinum (513); abscess of lung (513.0); abscess of mediastinum (513.1); pulmonary congestive hypostasis (514); postinflammatory pulmonary fibrosis (515); other alveolar and parietoalveolar pneumonopathy (516); pulmonary alveolar proteinosis (516.0); idiopathic pulmonary hemosiderosis (516.1); idiopathic fibrosing alveolitis (516.3); other specified alveolar and parietoalveolar pneumonopathies (516.8); unspecified alveolar and parietoalveolar pneumonopathy (516.9); lung involving other condition (517); rheumatic pneumonia (517.1); systemic sclerosis lung disease (517.2); lung involvement in other disease classified elsewhere (517.8); pulmonary collapse (518.0); pulmonary eosinophilia (518.3); acute lung edema not specified (518.4); post traumatic pulmonary insufficiency (518.5); acute bronchopulmony aspergillosis (518.6); acute respiratory failure (518.8); acute respiratory failure (518.81); other pulmonary insufficiency (518.82); chronic respiratory failure (518.83); acute and chronic respiratory failure (518.84); tracheostomy complications (519.0); tracheostomy complication unspecified (519.00); infection of tracheostomy (519.01); mechanical complication of tracheostomy (519.02); other tracheostomy complication (519.09); cyanosis (782.5); orthopnea (786.02); hemoptysis (786.3); respiratory arrest (799.1); tracheostomy status (V44.0); acquired absence lung

	(V457.6); dependence on respirator (V46.1); machine dependence supplemental oxygen (V46.2); attention to tracheostomy (V55.0)

A patient has high complexity COPD if he or she has been identified as having COPD and has any of the following diagnoses or services during the reporting period that are designated as high. A patient has moderate complexity COPD if he or she has been identified as having COPD, has not been designated as having high complexity COPD, and has had any of the following diagnoses or services during the reporting period that are designated as moderate.
dx, diagnosis; TB, tuberculosis
